# The Benefits of Merging Leadership Research and Emotions Research

**DOI:** 10.3389/fpsyg.2016.01022

**Published:** 2016-07-06

**Authors:** Ronald H. Humphrey, Gerald F. Burch, Laural L. Adams

**Affiliations:** ^1^Department of Leadership and Management, Lancaster University Management School, Lancaster UniversityLancaster, UK; ^2^Department of Management, Tarleton State University, StephenvilleTX, USA; ^3^School of Business, Virginia Commonwealth University, RichmondVA, USA

**Keywords:** leadership, emotions, affect, emotional labor, charisma, affective events, emotional regulation, transformational leadership

## Abstract

A closer merging of the literature on emotions with the research on leadership may prove advantageous to both fields. Leadership researchers will benefit by incorporating the research on emotional labor, emotional regulation, and happiness. Emotions researchers will be able to more fully consider how leadership demands influence emotional processes. In particular, researchers can better understand how the workplace context and leadership demands influence affective events. The leadership literature on charisma, transformational leadership, leader-member exchange, and other theories have the potential to shed light on how rhetorical techniques and other leadership techniques influence emotional labor, emotional contagion, moods, and overall morale. Conversely, the literature on emotional labor and emotional contagion stands to provide insights into what makes leaders charismatic, transformational, or capable of developing high quality leader–follower relationships. This review examines emotions and leadership at five levels: within person, between persons, interpersonal, groups and teams, and organizational wide and integrates research on emotions, emotional contagion, and leadership to identify opportunities for future research for both emotions researchers and leadership researchers.

## Introduction

Key researchers across a variety of theoretical approaches have recognized that leadership is inherently an emotional process. In particular, charismatic leadership attributes an important role to the leader’s ability to inspire followers and create a sense of a common identity (Conger and Kanungo, 1987; Conger et al., 2000; Conger, 2011). Inspirational, charismatic leadership may be especially critical during times of crisis (Halverson et al., 2004) or during times of great opportunities (Conger, 2011) when emotions are likely to be highly engaged. Transformational leadership researchers acknowledge that leaders need to be charismatic and inspirational if they want followers to buy into their visions (Bass and Riggio, 2006). Likewise, leader-member exchange theorists state that affect is important to leader–follower relationships (Schriesheim et al., 1999), and scholars developed leader-member exchange subscales to measure affect in terms of the amount of liking and friendship between leaders and followers (Liden and Maslyn, 1998). Hence, a wide variety of leadership theories and approaches attribute an important role to emotional components, suggesting the usefulness of establishing a greater linkage between research on emotion and leadership.

Researchers, who primarily study emotions tend to focus on internal affective experiences. As such, they have a great understanding of how emotions are regulated (Gross, 1998, 2006; Grandey, 2000). However, emotions tend to be stimulated by external events, and people are likely to encounter a large number of highly important and emotionally charged events at work. Affective Event Theorists (AET) have considered how workplace events influence employees’ emotions (Weiss and Cropanzano, 1996; Weiss et al., 1999). Researchers have applied AET to leadership, and this research shows great potential in expanding our understanding of the interrelationships between emotions and leadership. Research on AET and on emotions and leadership was stimulated by a special issue of *The Leadership Quarterly* edited by Humphrey (2002), and this interest has continued, as witnessed by the number of newly published articles on the topic (e.g., Griffith et al., 2015; Koning and Van Kleef, 2015). However, many questions about the relationships between emotions and leadership remain.

One area where progress has been made is the growing literature on how leaders use emotional labor tactics (e.g., Humphrey, 2008, 2012; Humphrey et al., 2008; Hunt et al., 2008; Gardner et al., 2009; Iszatt-White, 2009, 2012). Hochschild (1983, p. 7) was the first to study emotional labor, and she conceptualized emotional labor as the “management of feeling to create a publicly observable facial and bodily display.” Researchers have traditionally studied emotional labor in the service industry by observing how wait staff, retail employees, airline attendants, nurses and health care providers, and sales agents manage their emotional displays to “provide service with a smile” or to show emotions appropriate to their role (such as care and concern; see Grandey et al., 2013, for a comprehensive review of the literature). However, researchers can make additional strides in understanding the complex dynamics between leaders and emotions by leveraging the insights from emotions and leadership fields. Toward this aim, we merge insights from research on leadership, emotions, and emotional labor to better understand research gaps and opportunities that exist for both leadership and emotions researchers. We organize this review around a multi-level model of emotions and leadership.

## Multi-Level Model of Emotions and Leadership

Research has established that individuals, groups, and therefore, organizations, are pervaded by feelings (Menges and Kilduff, 2015), or emotions, which affect individuals and their organizations. These effects can have far-reaching impacts since emotions are transferrable between followers, from leader to follower, or from follower to leader. Finally, once these emotions are evident, they must be dealt with in social settings. We organize this review by drawing upon Ashkanasy’s (2003a,b) multilevel model of emotions in organizations as it was extended to leadership by Ashkanasy and Humphrey (2011a,b, 2013) and Humphrey (2013). This model examines emotions in organizations at five levels: (1) within person, (2) between persons, (3) interpersonal, (4) groups and teams, and (5) organizational. Below, we define some key terms before investigating each level.

There are many definitions for emotions (Gooty et al., 2010). Emotions are usually discussed at the individual level where an event, object, or affiliation elicit a short lived feeling of joy, happiness, fear, anxiety, sadness, pride, anger, guilt, or shame (Frijda, 2007; Izard, 2009; Menges and Kilduff, 2015). Cognitive Appraisal Theory defines emotions as organized mental responses to an event or entity (Ortony et al., 1988; Izard, 1991). Moods are less intense than emotions (Fisher, 2000, 2002; Gohm and Clore, 2002) and range from positive to negative, with no focal stimuli, and are often not consciously recognized by the individual (Watson and Tellegen, 1985; Frijda, 1993). The term ‘affect’ is often used as “the umbrella term that embraces both emotions and moods as well as other constructs with relevance to emotions” (Menges and Kilduff, 2015, p. 849). These terms can also be extended to the group level where group emotions are specific felt responses to specific stimuli or to the underlying mood of the group.

Included in the previous definitions is the understanding that emotions are triggered by events. Gooty et al. (2010) stated that the dominant emotions framework is built around Affective Events Theory (AET) where employees respond to discrete affective events in the workplace, which in turn lead to attitudinal or behavioral outcomes (Weiss and Cropanzano, 1996). However, it is possible for individuals to transfer their emotions to others that have not experienced the same event. Emotional contagion is defined as the “tendency to automatically mimic and synchronize facial expressions, vocalizations, postures, and movements with those of another person and, consequently, to converge emotionally” (Hatfield et al., 1994, p. 5). In organizations it is possible that this convergence can be within and between persons, as well as based on group or organizational processes (Tee, 2015).

Integrating these concepts, we propose that (1) emotions are present in the workplace, (2) employees may experience these events outside of work and bring their emotions to the workplace, (3) other employees (including leaders) can trigger emotional events during the day, (4) events that occur during the workday or are relived through memories while at work will create emotions, (5) these emotions must be dealt with, either through self-regulation or emotional labor, (6) emotions may be transferred to other employees, (7) leaders are susceptible to these same events, and (8) leaders are capable of influencing the emotions and moods of their followers (both positively and negatively). It is this pattern of feeling and responding that lies at the integration of leadership and emotions research. In the following sections we highlight the mutual benefits to emotions researchers and leadership researchers that can ensue from more closely integrating the two literatures.

## Level 1(Within Person): Fluctuations in Emotions and Emotional Reaction to Events

Emotions in Level 1 are often associated with AET (Ashkanasy and Humphrey, 2011a) since this level is concerned with how an individual’s emotions fluctuate moment to moment in response to various environmental events. AET provides a model of how people’s moods and emotions vary from their normal baseline level of affect according to workplace events (Weiss and Cropanzano, 1996; Weiss et al., 1999). Positive events, called “uplifts,” such as a pleasant interaction with a customer or coworker, can elevate people’s mood above their normal baseline. In contrast, negative events or “hassles” can lower an employee’s mood below the individual’s baseline. In addition, biorhythms influence how emotions and moods vary throughout the day. Thus, this level involves the ability of leaders and followers to maintain their energy, enthusiasm, and positive spirits throughout the workday. Psychologists have spent considerable time studying how people can retain and improve their levels of affect, and this research could significantly benefit leadership researchers in studying how leaders maintain their own energy stores.

In recent years, researchers, practitioners, and the public have all become increasingly interested in how people can feel happy and preserve their positive emotions (e.g., Fredrickson, 2001; Lyubomirsky, 2008). Researchers have discovered how positive emotions can improve performance and creativity through a “broaden and build” process that leads to greater access to cognitive resources (Fredrickson, 2001). They have also developed a variety of exercises and “happiness activities,” such as gratitude exercises, that have been shown to improve levels of positive affect (Lyubomirsky, 2008). It is likely that this literature can help leadership researchers understand how leaders can better manage their within-person fluctuations in emotions and energy. However, relatively little research has been done to see if these happiness activities can translate into workplace settings and improve leadership performance, so this area is rife with opportunities for innovative research.

Much of the happiness literature derives from the broader positive psychology movement, and some leadership researchers have taken a positive psychological approach to leadership. For example, Luthans et al. (2007) and Hannah and Luthans (2008) have examined the role of positive psychological capital (i.e., confidence and self-efficacy, hope, optimism, and resiliency) in determining leadership effectiveness and resilience to environmental stressors. It is likely that self-efficacy (Kellett et al., 2009) and related psychological capital variables can help leaders maintain positive mood levels while encountering typical workplace frustrations and even crisis situations.

A second area for consideration in Level 1, which concerns emotions within the person, is the means by which emotional contagion is transferred indirectly from other individuals through affective events. Tee (2015) proposed that emotional contagion processes are either automatic or deliberate. It is therefore possible for any group member to intentionally, or unintentionally, create events where their emotions are transferred to other members. Leaders, who understand these fluctuations in emotions and how individuals regulate emotions are more capable of managing these processes across the organization. More discussion about this is offered in Levels 3 and 4. However, research in emotions has shown that the measures used to evaluate emotional responses matter (Mauss and Robinson, 2009). There is no “gold standard” by which researchers can measure emotional responses, so they must carefully choose from among the experiential, physiological, and behavioral alternatives available when investigating emotions and the process of emotional contagion.

Another consideration in Level 1 is that daily events will often cause employees to behave impulsively (Ashkanasy and Humphrey, 2011a), which can result in the need for emotional labor to complete the day’s tasks. Researchers have discovered that some forms of emotional labor are more beneficial than others for maintaining positive affect. Hochschild (1983) described two forms of emotional labor: surface acting (faking emotional expressions) and deep acting (deliberately trying to feel the emotions one is supposed to display). Later scholars argued that a third form of emotional labor – natural, genuine, and spontaneous emotional labor – is also a valid form (Ashforth and Humphrey, 1993; Glomb and Tews, 2004; Diefendorff et al., 2005; Dahling and Perez, 2010). Natural emotional labor occurs whenever people’s spontaneous emotions are appropriate for their role and situation and often arises when people identify with their roles.

The final and important point at this level that leaders are “human too.” As such, leaders are susceptible to alterations in emotions and moods based on daily activities and interactions with others. Ilies et al. (2005) suggested that followers with high positive affect can influence leader’s emotions through emotional contagion. This creates a bi-directional emotional contagion that stands to improve the workplace environment (Rajah et al., 2011). Emotions research may be beneficial in this area, particularly since some people are more aware of their emotions, more susceptible to emotional stimuli and have stronger mood effects on their judgment (Gasper and Clore, 2000; Gohm, 2003; Marroquín et al., 2016). Individual differences can therefore cause leaders to alter their leadership style. Collins and Jackson (2015) showed that leaders who were exposed to high levels of emotion affected their self-regulation and resulted in destructive leadership. Conversely, lowering the level of negative emotions allowed leaders to self-regulate more effectively, giving rise to constructive leadership. Researchers have sought to understand how emotions effect leader decision-making (Ashkanasy and Daus, 2002; Humphrey, 2002), but very little research has been conducted in this area. Additional investigation would benefit both leadership researchers and emotions researchers.

### Benefits to Leadership Researchers

Benefits to leadership researchers include (1) an understanding of how emotional labor strategies can help leaders manage their energy throughout the day, as well as whether and how various emotional labor strategies can boost leader well-being or increase stress; (2) a recognition that leaders are susceptible to the same emotional triggers as followers; and (3) the realization that emotional contagion can be both a positive and negative contributor to Level 1 emotions in an organization.

### Benefits to Emotions Researchers

Benefits to these researchers include (1) a better understanding of the affective events that influence emotional labor, as well as actors’ emotions and their choice of emotional labor tactics and (2) the understanding that organizations are filled with emotions that must be managed in order to complete organizational tasks.

## Level 2 (Between Persons): Individual Differences

Level 2 concerns how individuals uniquely regard and choose to interact with others, as well as the ways in which these individual differences impact emotions; it involves the leader’s emotionally informed perspective of others. In this level, “individual differences determine frequency, intensity, and duration of the experience of positive and negative moods and emotions” (Ashkanasy and Humphrey, 2011b, p. 216). Leadership researchers have extensively examined personality traits, as is evidenced by the fact that most comprehensive leadership textbooks have chapters devoted to personality (e.g., Humphrey, 2013). Leadership researchers in particular have studied the Big Five personality traits in considerable depth. For example, see the classic meta-analysis by Judge et al. (2002). Although all of the Big Five traits are individually related to performance, only extraversion and openness are related to leadership effectiveness when considered together in regression analysis. The Big Five have been studied with regard to emotional labor, and the overlap in effects is considerable and consistent. The Wang et al. (2011) meta-analysis found that extraverts were more likely to use deep acting, and less likely to use surface acting. In addition, they found that neuroticism was also negatively related to deep acting and positively associated with surface acting, which suggests that leaders with this trait employ surface acting strategies to manage emotions in organizational contexts.

Leadership researchers, however, have gone far beyond an exclusive focus on the Big Five. For example, Gardner et al. (2009) proposed that emotional intelligence, self-monitoring ability, and political skill all moderated leaders’ emotional displays, the visible signs of emotions, to events that are affective in nature. This is supported by a wide-ranging meta-analysis that examined a total of 25 distal (personality traits) and proximal (state-like developed attributes) characteristics (Hoffman et al., 2011). However, few studies have examined individual traits that impact emotional labor beyond the Big Five.

Although emotional labor researchers would seem to have the edge when it comes to studying emotional intelligence, both fields have actively investigated the construct and found that emotional intelligence is beneficial to individual employees and organizations. Among leadership researchers, Boyatzis et al. (2011) found that emotional competency is related to a variety of positive outcomes. Walter et al. (2011) reviewed the literature and concluded that emotional intelligence is related to emerging as a leader, the performance of effective leadership behaviors, and overall leader effectiveness. Likewise, the meta-analysis by Wang et al. (2011) found that emotional intelligence is positively correlated with the deep acting, the form of emotional labor that aligns with an individual’s identity and role expectations, and negatively correlated with surfacing acting, the stress-producing form of emotional labor.

Another consideration at this level is that leaders have individual differences that may make them more susceptible to emotional contagion (Tee, 2015). These individual differences may include the leader’s empathy, extraversion, neuroticism, and overall susceptibility to emotional contagion. Additionally, individual differences in affective forecasting play a role in the between persons level. Emotions researchers Hoerger et al. (2012) have examined the role of emotional intelligence in people’s ability to accurately predict reactions to emotionally charged events. Researchers working in leadership would benefit from exploring these areas too.

Outside of the emotional labor field, it is likely that emotions researchers have explored a wider range of the personality traits and competencies that allow people to manage their emotions and their emotional displays. Thus, there are benefits to researchers in both fields from a closer integration of the two.

### Benefits to Leadership Researchers

The benefits to those studying emotions through the leadership perspective would include (1) a deeper understanding of the differences in individual ability to regulate emotions that could help explain differences among leaders, (2) a deeper appreciation for the potential role of individual differences on leaders’ susceptibility to emotional contagion, and (3) insights into the individual differences that may influence leaders’ abilities to perform emotional labor and respond effectively to affective events in the workplace.

### Benefits to Emotions Researchers

The benefits that emotions researchers stand to gain from incorporating leadership literature into their work include (1) a consideration of a wider range of personality traits and competencies that might influence actors’ abilities to perform emotional labor, and (2) an understanding of how emotional intelligence, political skill, neuroticism, and extraversion may influence individual susceptibility to emotional contagion.

## Level 3 (Interpersonal): Emotional Contagion, Dyadic Leadership, and Emotional Labor

Effective organizations rely on good communication, but it is important to recognize that an effective leaders does not simply engage in one-on-one communication with another organizational member. Instead, the communication is bi-directional. Level 3 addresses this dyadic communication and the role that emotion plays in the transfer of ideas and feelings. This level considers emotions as a form of communication, as well as how the receiver makes appraisals of the message and sender and the effect this communication has on others based on the emotion shaping it. This level also entails the ways in which emotional labor can be utilized in interpersonal relationships.

Leaders are responsible for communicating the required tasks to followers and for being “manager(s) of group emotions” (Pescosolido, 2002). As such, regulation of group member emotions is an important leadership function (George, 2000; Humphrey, 2002). However, empirical studies have shown that the emotional expression by leaders is often more important than the content of the message (Newcombe and Ashkanasy, 2002) since leaders influence follower attitudes, cognitions, affective states, and behavior (Koning and Van Kleef, 2015). Emotional expressions are therefore powerful tools of social influence (Côté and Hideg, 2011; Van Kleef et al., 2011) that transfer information about feelings, attitudes, and intentions toward others (Koning and Van Kleef, 2015), either by affective reactions or through inferring then adopting the emotions of others (Van Kleef, 2009; Van Kleef et al., 2009).

Affective reactions include processes like emotional contagion. Emotional contagion processes are essential for effective leadership (Tee, 2015), particularly when leaders deliberately try to induce and spread emotions through a group. Similarly, these same processes can influence a wide range of team and leader outcomes when emotional contagion moves from the follower to the team or the leader (Tee, 2015). However, the leader’s intentional message may not always be received the same by the follower since the receiver’s inferential processes may have a significant role in appraising and interpreting the message (Rajah et al., 2011). Studies by Eberly and Fong (2013) showed that followers share leaders’ emotions and attribute sincerity, or a lack of it, to leaders’ intentions. These attributions then affect followers’ perceptions of their leaders’ effectiveness. Some of these attributions may be based on things the leader cannot control, such as gender, for example. Schaubroeck and Shao (2012) empirically demonstrated that female leaders who displayed sadness were more negatively evaluated than males who demonstrated sadness.

Another consideration for the role of emotions in dyadic situations, where communication is bidirectional, is the effect that it can potentially have on followers. Research has shown that when a leader praises a follower for a job well-done, the effects are positive. Such acknowledgment makes recipients feel valued and serves as open recognition of followers’ efforts in overcoming difficult tasks (Iszatt-White, 2009). Early studies of emotions showed that leaders higher in positive affect had service workers that were more willing to help customers, sold more products, and had lower turnover rates than leaders who ranked lower in positive affect (George and Bettenhausen, 1990). This early work on the role of positive affect in dyadic relationships has given rise to more recent research aimed at unraveling the complexities of leaders’ emotional displays and followers’ reactions.

The research suggests that, at the more basic level, leaders who demonstrate positive emotions have followers that attribute their intentions positively (Dasborough and Ashkanasy, 2002). Similarly, negative emotions have been seen to lower follower creative performance (Visser et al., 2013). However, the reason for the response and the type of response seem to matter to the follower. Madera and Smith (2009) found that in a crisis situation, leaders who expressed sadness were evaluated more favorably than those who exhibited anger. Other studies have shown that happy displays by the leader increased follower creative performance while sad displays increased follower analytical performance (Visser et al., 2013). These responses may also be moderated by the type of industry where positive emotions may have more influence in industries requiring high degrees of emotional labor (Johnson, 2008).

Follower reactions are important for two reasons. First, a follower’s initial reaction may influence many important organizational variables in the short term. Second, emotional events created by the leader may affect the relationship between the leader and the follower, which can have long-term impacts. Our review has shown that emotional displays can affect sales, creative performance, and analytical performance. Other studies have shown that emotional displays affect employee engagement (Lu and Guy, 2014) and organizational citizenship behaviors (OCB; Koning and Van Kleef, 2015). These are important short-term effects, yet, the long-term effects of emotional displays may be considerably more significant.

Kacmar et al. (2012) investigated the relationship between members’ OCBs and conflict in their relationships with bosses, observing that supervisor trust was a mediator. Based on these results it is expected that a leader’s display of emotions could give rise to relationship conflict, which in turn could lower a follower’s trust in the leader and potentially affect the follower’s appraisal of future emotional displays. Such actions could then cause long term effects in individual task performance and OCBs. This may be especially important in situations where the leader must correct the follower’s behavior or address performance issues. It is clear that, in cases like this, leaders do not only respond with positive emotions, however, little research has been conducted on leaders’ use of neutral or negative emotions to effectively alter employee performance. This suggests that certain emotions are more appropriate than others in particular contexts, and the use of appropriate emotional displays will have a pivotal role in building rapport between follower and leader (Koning and Van Kleef, 2015), even if the emotion is anger. These dynamics may affect followers’ appraisals of leaders; further, events may cause different emotions in followers when similar emotions lead to different outcomes and when different emotions have the same indicators (Rajah et al., 2011).

Further complicating the task of unraveling the role of emotions in organizations is the observation that emotions do not occur in isolation. More than one emotion can be felt at a time, as followers report feeling different levels of anger, pride, and shame based on the leader’s emotional expression and the follower’s perception of the appropriateness of the expression (Koning and Van Kleef, 2015). This discussion of emotions as communication, emotional contagion, and the appraisal of emotional expressions underscore the importance of emotions in Level 3 of an organization. The literature reveals leaders as emotion managers and this regulation as having significant consequences for both leaders and followers. We now turn to how these emotions can be managed.

Emotional labor is by its very nature concerned with the interaction between two people, the service agent and the client. However, given this focus, it is somewhat surprising how little of the emotional labor literature focuses on the dyadic interactions between the two. Instead, most of the literature has examined how emotional labor affects the well-being of the service agent. Relatively few have even examined how emotional labor affects the perceptions of service quality (see the meta-analysis by Hulsheger and Schewe, 2011). Thus, emotional labor research could benefit greatly by considering the interpersonal influence tactics used by leaders, as well as the general interpersonal interactions between leaders and individual followers. Among leadership researchers, leader-member exchange researchers have specifically focused on the interpersonal one-on-one dyadic interactions between followers and leaders (Dansereau et al., 1975). Meta-analyses have supported the role of the leader-member model for explaining leader performance. Gerstner and Day (1997) found support for the relationship with performance, as well as with subordinate job satisfaction. Likewise, Ilies et al. (2007) found that leaders with high quality relationships with followers have followers who perform more OCBs. Researchers have examined how a wide variety of processes, such as emotional displays and attributional processes, influence the relationship between followers and these types of behaviors (e.g., Dasborough and Ashkanasy, 2002). This line of research provides a model for the type of variables that might also influence the interpersonal interactions between emotional labor agents and their clients or patients.

In addition to the under-explored area of dyadic interactions between the service agent and recipient is the link between an emotional labor provider’s charisma and the emotional interactions that ensure. However, researchers who focus on leadership have given considerable attention to charisma (Conger and Kanungo, 1987; Conger et al., 2000; Conger, 2011). Likewise, transformational leadership researchers ascribe considerable importance to charisma (Bass and Riggio, 2006). It is easy to presume a strong link between emotional laborers’ charisma and emotional labor interactions, yet to our knowledge the connections between a service provider’s charisma and the emotional interactions with the client have never been examined. Leadership researchers have also explored how crisis situations influence attributions of charisma (e.g., Halverson et al., 2004). Emotional labor has frequently been studied in settings, such as health care (e.g., emergency rooms) or law enforcement, where crisis situations are common, so it makes sense to integrate these two bodies of knowledge.

Charismatic researchers attribute a considerable amount of charisma to rhetorical skills, and they have studied how specific techniques, such as the use of metaphors and “we” statements, contribute to perceptions of charisma (Emrich et al., 2001; Mio et al., 2005; Murphy and Ensher, 2008; Naidoo and Lord, 2008). These researchers have found that rhetorical skills such as these can create an emotional reaction in their followers. Given that the whole purpose of emotional labor is to create an emotional impression in the receiver, it is somewhat surprising that leadership researchers have not studied the relationship between rhetorical skills and emotional labor, as the results could significantly extend our understanding of the dynamics of emotional labor.

Conversely, the advantages of informing research with knowledge from other disciplines do not all accrue only to leadership researchers, as there are some gaps in emotions research as well. In particular, emotions researchers generally have not examined how portraying emotions creates an emotional impact, sometimes quite draining, on the speaker. Instead, rhetorical skills are largely portrayed as a cognitive skill, and the emotional impact on the leader tends to be overlooked. This omission is quite puzzling given that it is well known that many people have a fear of public speaking, and that speaking in front of groups can be quite intimidating. This is an area in which emotional labor researchers excel, for they have extensively studied how portraying emotions can have deleterious effects on emotional laborers (Hulsheger and Schewe, 2011; Grandey et al., 2013). Although performing emotional labor can have harmful effects on actors’ well-being (Grandey et al., 2015), this is likely to be true when there is poor person-job fit, or when the actors use the negative form of emotional labor (surface acting) instead of the more beneficial forms (deep acting and genuine, natural emotional labor, Humphrey et al., 2015). Indeed, when emotional laborers identify with their role and express emotions consistent with their identity, this may even have beneficial effects. The benefits may be especially true for leaders, who, like stage actors, may get a thrill from being in front of others. Thus, as in Level 1, the literature suggests that the effective management of emotional labor may be key to whether leaders experience expressing emotion as harmful or beneficial to their well-being.

The effective management of emotional displays through emotional labor may also strongly determine whether leaders are successful or not in establishing productive one-on-one relationships. Research has shown that leaders’ emotional displays play a large role in subordinates’ attributions about the leaders’ sincerity (Dasborough and Ashkanasy, 2002). Although leaders may be sincerely well-intentioned toward their subordinates, normal workplace frustrations, and hectic days may make it difficult for leaders to portray the appropriate emotions when dealing with subordinates, even if they can summon up the appropriate words (Humphrey, 2008; Humphrey et al., 2008). Research suggests that such surface acting may often produce bad impressions on others, because observers can often detect the actors’ true emotions leaking through, and thus they perceive the portrayed emotions as fake and insincere. Thus, a leader’s selection of emotional labor tactics, such as opting to surface act positive emotions or choosing to reveal true feelings even if they are negative, may significantly impact their ability to establish high quality trusting relationships with their followers. Research in these areas could afford benefits to both leadership researchers and emotions researchers.

### Benefits to Leadership Research

From a merger between leadership research and emotions research, leadership researchers stand to gain (1) a better understanding of how the choice of emotional labor strategies influences the quality of the one-on-one relationships between leaders and followers, (2) the importance of further unraveling followers’ appraisals of leaders’ emotional displays, (3) a deeper understanding of the link between emotional displays and relationship conflict, and (4) the recognition that there is more to discover regarding types of emotions and their subsequent organizational outcomes.

### Benefits to Emotions Researchers

Such a merger of literatures would enable emotions researchers to gain (1) a more in-depth understanding of the context in which emotional labor between leaders and followers takes place, (2) the role of gender in appraisal of emotions, and (3) additional understanding of the role of rhetoric in emotional labor, including the use of pronouns, metaphors, word choice, etc.

## Level 4 (Groups and Teams): The Locus of Emotional Contagion

In contrast to Level 3, which considers the dyadic interaction between a leader and a follower, Level 4 attempts to explain the effects of emotions at the group level by evaluating the direct influence of the leader on the group and the subsequent changes to the group’s affect and performance. Much leadership takes place at the group level. However, a study of empirical leadership research shows that significantly less attention has been devoted to team and unit-level processes and outcomes (DeChurch et al., 2010).

Of the research that has examined emotions at the group level, researchers have shown how leaders can influence emotional contagion among group members (e.g., Bono and Ilies, 2006). Groups and teams are often composed of many members who all have the capacity to transfer their emotions to the group. In fact, it is the development of group emotion that defines a group and distinguishes it from a collection of individuals (Barsade, 2002). “Group emotion does not average out the individual team members’ score to arrive at the output” (Vijayalakshmi and Bhattacharyya, 2012). Instead, there is a synergistic affect that leads to collective emotion as a result of contagion between group members (Bartel and Saavedra, 2000; Barsade, 2002) or between the group’s leader and its followers (Sy et al., 2005). The emergence of such “affective tones” (George, 1990, 1996) tend to be group-level phenomena (Menges and Kilduff, 2015).

The emotional contagion process at this level is apparent in the sharing and transferring of emotions, resulting in an overall group-level affect (Tee, 2015) and may lead to group identity. Often times traumatic events, such as the Madrid terrorist bombings, lead to collective emotions amongst groups that partially identify group membership (see Conejero and Etxebarria, 2007). The result is that emotional contagion in groups may enhance solidarity between group members that shapes group identity and motivates collective action (Tee, 2015). Conversely, it has been proposed that negative emotions from the leader may spread through the group which may negatively affect the quality of the group-level team member exchange relationship (Dasborough et al., 2009).

Recent research has made distinctions between group-shared emotions and group-based emotions (Menges and Kilduff, 2015). Group-shared emotions are those emotions that members collectively experience during interactions with other group members. Group-based emotions are experienced individually and are based on group membership, absent of other group members (Bar-Tal et al., 2007; Niedenthal and Brauer, 2012). This distinction is important since it could explain how individuals in a group may evaluate emotional events in different ways given different origins of the group’s emotions.

A second distinction made by Menges and Kilduff (2015) is that group emotions may converge or diverge. Current research has focused on emotional convergence where group members feel similarly, often based on emotional contagion. However, it is possible, and even likely, that all group members do not feel the same. This is witnessed in research where individuals in the most powerful positions in the group tend to feel differently than those who occupy positions of lower power (Kemper, 1990, 1991; Tiedens et al., 2000). It is also possible that some members of the group will have individual divergences in feeling, but that the group maintains an overall convergence in emotions (Menges and Kilduff, 2015). Group emotional divergence and convergence is important to leaders for two reasons. First, the leader may not be feeling the same emotion as the followers. Second, a leader’s appropriately timed emotional display may result in members receiving the message differently, depending on the group’s lack of emotional synchrony and homogeneity.

Similar to the process by which followers appraise leaders’ emotional displays at the individual level, it appears that groups may determine the valence they apply to leaders’ emotions. Research in crisis situations, similar to the Madrid bombings, show that emotional contagion from leaders to followers is stronger during crisis situations (Madera and Smith, 2009). Conversely, more autonomous teams may have fewer opportunities for the leader to display emotions, which can result in lower follower-trust relationships (Tee, 2015) and lower levels of leader-induced emotional contagion. An example of this appraisal is seen in the study by Zampetakis and Moustakis (2011), where researchers observed that a group’s evaluative judgment of their leader’s emotional intelligence mediated the relationship between the leader’s *actual* emotional intelligence and the group’s job satisfaction measures.

Another consideration for leaders in managing group affect is that followers get to determine whether the emotional display is appropriate. Koning and Van Kleef (2015) empirically demonstrated that follower willingness to perform OCBs was decreased by their leader’s display of anger, and that these effects were magnified when the anger was deemed inappropriate by the follower. However, appropriate displays, for example, toward an issue, person, or organization – ones in alignment with the followers’ or the group’s emotions – may play a pivotal role in building rapport between leaders and groups. Such displays may unite teams against common enemies or issues. It therefore appears that the appropriateness of the display and the target of the emotional display may be relevant in leading teams.

However, from the vantage of emotions research, there are relatively few emotional labor studies at the group level. A notable exception is the study on how group emotional labor norms are developed among nurses (Diefendorff et al., 2011). Thus, there seems to be considerable potential for emotional labor researchers to gain insights into emotional labor by studying the leadership of teams and groups. One area that may yield significant insights is research that assesses leaders’ role in managing the cognitive appraisal of events (Pirola-Merlo et al., 2002), a role they are afforded in groups because they are believed to have access to relevant, privileged information and possess the knowledge and skills to make sense of events (Smircich and Morgan, 1982; Gioia and Chittipeddi, 1991; Maitlis, 2005; Menges and Kilduff, 2015).

Although leadership researchers have studied group emotional contagion processes, they still need to examine how emotional labor tactics influence group processes. Case studies suggest that leaders’ emotional labor can have powerful effects on group members, for good or ill, and that the effects of leader emotional displays can cause a chain reaction that ripples throughout the organization (Burch et al., 2013). However, this needs to be explored empirically using larger samples. Such exploration has the potential to benefit both leadership researchers and emotions researchers.

### Benefits to Leadership Research

The merging of findings from emotions research with those of leadership research would yield (1) greater insight into how the choice of emotional labor strategies influences group cohesion, morale, and emotional contagion processes, (2) the realization that group emotions may not be homogenous, and (3) an appreciation of the role of group-shared versus group-based emotions at the group level.

### Benefits to Emotions Researchers

Merging research from emotions and leadership would benefit emotions researchers by providing (1) a novel approach through which to understand emotional labor processes at the group level, and (2) a vast amount of yet untapped literature that can aid in developing theory at this level.

## Level 5 (Organization-Wide): Organizational Culture and Emotional Labor Display Rules

It is clear that leadership at the top of an organization is important, not only for setting the strategic direction of the company, but also for creating an organizational culture that gives the organization its unique organizational identity. It has been argued that humans complete themselves through culture (Geertz, 1973) and the new marketplace of ideas has allowed many organizations the opportunity to provide a culture that is attractive to its members (Rafaeli and Worline, 2001).

There are a variety of ways in which organizations create their organizational culture and get their members to identify with the organization. Organizations use distinctive clothing and fashion trends that distinguish their organizational members from other groups, and that can also help communicate a member’s organizational place in the hierarchy (Dutton et al., 1994; Pratt and Rafaeli, 1997). Flags, mascots, specialized insider jargon, and even architecture and office design all operate to generate an organization’s unique culture and identity (Pratt and Rafaeli, 2001). However, research also suggests that the interactions between people are bound by rituals and routines that function as symbols with the potential to infuse group members with more emotions than other cultural artifacts can do (Collins, 1990, 2004, 2014).

Distinctive clothing, cultural artifacts, the basic sequence of greeting members, gathering in common places and interaction rituals may all play an important role in supporting emotional labor since they create a joint focus of attention and a coherent, synchronized emotional energy. Likewise, the reverse is also likely to be true; namely, emotional labor can likely impart cultural artifacts with their meaning. “A person’s feelings, then, are determined not only or even primarily by internal individual characteristics, but by things such as organizational structure, hierarchy and status, organizational design, organizational leadership, and organizational culture” (Rafaeli and Worline, 2001, p. 105). Thus both areas may benefit from a closer integration.

What results from the dynamics of emotions at this level of the organization is the development of an affective climate. Choi et al. (2003) define “affective climate” as an overall interaction pattern or a shared positive perception among members, and the atmosphere that characterizes interaction within a team. Characteristics of this climate serve as social control mechanisms that guide how team members interpret events, develop attitudes, and develop expectations about their behaviors (De Rivera, 1992; Dasborough et al., 2009). More recent research has considered the negative impact that individual members’ negative emotions may have on the affective climate (Dasborough et al., 2009).

This evidence of emotions at the organization-wide level strongly suggests a role for emotional contagion processes operating at this level since implicit and explicit emotional contagion processes allow for the spread of emotions across social networks (Tee, 2015). This sharing of emotions therefore shapes and revises the culture of the organization, a process that implicates leaders’ roles in managing organization-level emotions. Given the spreading of emotions across organizations, leaders should carefully consider how their culture encourages and facilitates the appropriate display of leader emotions.

Displays are deemed appropriate in relation to a group’s social conventions. Social regulations and norms affect individuals’ expression of emotions, prescribing that members suppress some emotions and openly display others (Sutton and Rafaeli, 1988). According to emotional labor theory, organizations create emotional labor display rules that specify the emotions that organizational members should display (Hochschild, 1983; Rafaeli and Sutton, 1987, 1989; Ashforth and Humphrey, 1993). Despite this clear theoretical statement about the creation of organizational norms, the research has remained intensely focused on individual reactions to emotional labor demands rather than on the repercussions of emotions rules at the organizational level. Importantly, Diefendorff and Greguras (2009) showed that organizational display rules for positive emotions resulted in individuals claiming to experience emotions on par with or at a slightly less intense register than the convention prescribed, while individuals often ignored the display rules for negative emotions (Schneider et al., 2014). However, a problem researchers have yet to address is the difficulty of empirically separating *felt* versus *displayed* emotions (Rajah et al., 2011). Overcoming this challenge, in part through merging leadership and emotions research, will create considerable opportunities for additional research at this level.

### Benefits to Leadership Research

The benefits for leadership researchers derived from merging the two literatures will include (1) an appreciation for how organizational emotional labor display rules are crucial to creating organizational culture, (2) a recognition of the importance of teasing out felt versus displayed emotions, and (3) an awareness of the role of individual emotions on organizational culture.

### Benefits to Emotions Research

The benefits accrued to emotions researchers by a merger of the two research fields includes (1) an understanding of how organizational rituals, stories, and other cultural artifacts aid in the development of organizational labor display rules and facilitate the performance of emotional labor and (2) insights into the role of the marketplace of ideas in aiding companies to define a culture that is attractive to employees.

## Conclusion

This review illustrates the value of merging leadership and emotions literatures. The statement that emotions are present in the workplace has been established through empirical research and leadership, and emotions researchers have acknowledged such. It is also safe to say that most researchers agree that employees experience emotional events outside of work and bring their emotions to the workplace. However, little research has been done in this area. It is possible that even something as simple as the loss of a football match may alter the overall emotional climate if the majority of a team or organization supports that football team. These events occur daily and understanding them would benefit both leadership and emotions researchers.

Our review has also shown that other employees can trigger emotional events during the day. Leaders, who can create events though dyadic contact and also through the implementation of policy that may be evaluated positively or negatively by the group, are certainly likely to be a frequent triggers of such events. For example, employees experience a rise in emotional feeling when they receive word that their leader has approved bonuses for them. Conversely, policies that increase workload, extend working hours, or remove privileges may have negative effects on followers even though they do not have direct dyadic contact in the delivery of the message.

Another consideration that we have worked to explore in this review is that when events occur, or are relived, during the workday, they create emotions for the employee. These emotions are real and affect how employees experience the workday. The literature review on happiness provides some possible ways for organizations to help manage these emotions during the day. However, additional avenues exist for leaders to raise the organization’s level of emotional intelligence, such as, for example, by noticing when colleagues or followers have experienced a negative event, since these emotions must be grappled with, either through self-regulation or emotional labor.

This review has also discussed at each level the processes whereby individual emotions may be transferred to other employees through emotional contagion. Our review found that leaders are susceptible to emotional contagion through their followers. Leaders are not immune to changes in emotions that occur throughout various levels of the organization during the workday. Our evaluation of individual differences will hopefully lead to research that investigates the effects of emotions that are derived from leaders’ individual differences and leaders’ susceptibility to emotional contagion via their role in the organization.

A final contribution of this review is our discussion of the role of the leader in managing the emotions and moods of followers. The discussion in the area of emotional labor is particularly suited for an integration with the leadership field. Unlike much of the research on emotions, this review focused on emotions that occur in the workplace, and we recognize that organizational members often regulate their emotions to fit the organizational environment. Further research on the development and implementation of organizational display rules is needed to understand organizational members’ underlying reasons for accepting or rejecting these rules. Finally, we end where we began, with the acknowledgment that leadership research relies heavily on understanding the emotions of individuals, while emotions researchers benefit greatly from understanding the mechanisms by which leaders impact others’ emotional trajectories.

## Author Contributions

RH wrote the initial draft as a sole author and contributed roughly half of the resulting paper’s overall value. He then added two authors to complete the revisions and final draft. GB took the lead on the revision and contributed roughly 40% and LA contributed the remaining 10% of the article’s final value.

## Conflict of Interest Statement

The authors declare that the research was conducted in the absence of any commercial or financial relationships that could be construed as a potential conflict of interest.

## References

[B1] Ashforth B. E. Humphrey R. H. (1993). Emotional labor in service roles: the influence of identity. *Acad. Manage. Rev.* 18 88–115. 10.5465/AMR.1993.3997508

[B2] Ashkanasy N. M. (2003a). “Emotions in organizations: a multilevel perspective,” in *Research in Multi-Level Issues: Multi-Level Issues in Organizational Behavior and Strategy* Vol. 2 eds Dansereau F. Yammarino F. J. (Oxford: Elsevier), 9–54.

[B3] Ashkanasy N. M. (2003b). “Emotions at multiple levels: an integration,” in *Research in Multi-Level Issues: Multi-Level Issues in Organizational Behavior and Strategy* Vol. 2 eds Dansereau F. Yammarino F. J. (Oxford: Elsevier), 71–81.

[B4] Ashkanasy N. M. Daus C. S. (2002). Emotion in the workplace: the new challenge for managers. *Acad. Manage. Exec.* 16 76–86. 10.5465/AME.2002.6640191

[B5] Ashkanasy N. M. Humphrey R. H. (2011a). “A multi-level view of leadership and emotions: leading with emotional labor,” in *The Sage Handbook of Leadership*, eds Bryman A. Collinson D. Grint K. Jackson B. Uhl-Bien M. (London: Sage Publications), 363–377.

[B6] Ashkanasy N. M. Humphrey R. H. (2011b). Current emotion research in organizational behavior. *Emot. Rev.* 3 214–224. 10.1177/1754073910391684

[B7] Ashkanasy N. M. Humphrey R. H. (2013). “Leadership and emotion: a multi-level perspective,” in *Oxford Handbook of Leadership and Organizations*, ed. Day D. V. (Oxford: Oxford University Press).

[B8] Barsade S. G. (2002). The ripple effect: emotional contagion and its influence on group behaviour. *Adm. Sci. Q.* 47 644–675. 10.2307/3094912

[B9] Bar-Tal D. Halperin E. De Rivera J. (2007). Collective emotions in conflict situations: societal implications. *J. Soc. Issues* 63 441–460. 10.1111/j.1540-4560.2007.00518.x

[B10] Bartel C. A. Saavedra R. (2000). The collective construction of work group moods. *Adm. Sci. Q.* 45 197–231. 10.2307/2667070

[B11] Bass B. M. Riggio R. E. (2006). *Transformational Leadership*, 2nd Edn Mahwah, NJ: Lawrence Erlbaum.

[B12] Bono J. E. Ilies R. (2006). Charisma, positive emotions, and mood contagion. *Leadersh. Q.* 17 317–334. 10.1016/j.leaqua.2006.04.008

[B13] Boyatzis R. Brizz T. Godwin L. (2011). The effect of religious leaders’ emotional and social competencies on improving parish vibrancy. *J. Leadersh. Organ. Stud.* 18 192–206. 10.1177/1548051810369676

[B14] Burch G. F. Humphrey R. H. Batchelor J. H. (2013). How great leaders use emotional labor: insights from seven corporate executives. *Organ. Dyn.* 42 119–125. 10.1016/j.orgdyn.2013.03.005

[B15] Choi J. N. Price R. H. Vinokur A. D. (2003). Self-efficacy changes in groups: effects of diversity, leadership and group climate. *J. Organ. Behav.* 24 357–371. 10.1002/job.195

[B16] Collins M. D. Jackson C. J. (2015). A process model of self-regulation and leadership: how attentional resource capacity and negative emotions influence constructive and destructive leadership. *Leadersh. Q.* 26 386–401. 10.1016/j.leaqua.2015.02.005

[B17] Collins R. (1990). “Stratification, emotional energy, and the transient emotions,” in *Research Agendas in the Sociology of Emotions*, ed. Kemper T. D. (Albany, NY: State University of New York Press), 27–57.

[B18] Collins R. (2004). *Interaction Ritual Chains.* Princeton, NJ: Princeton University Press.

[B19] Collins R. (2014). “Interaction ritual chains and collective effervescence,” in *Collective Emotions*, eds von Scheve C. Salmela M. (Oxford: Oxford University Press), 299–311.

[B20] Conejero S. Etxebarria I. (2007). The impact of the Madrid bombing on personal emotions, emotional atmosphere and emotional climate. *J. Soc. Issues* 63 273–287. 10.1111/j.1540-4560.2007.00508.x

[B21] Conger J. A. (2011). “Charismatic leadership,” in *Sage Handbook of Leadership*, eds Bryman A. Collinson D. Grint K. Jackson B. Uhl-Bien M. (Thousand Oaks, CA: Sage Publications), 86–102.

[B22] Conger J. A. Kanungo R. N. (1987). Toward a behavioral theory of charismatic leadership in organizational settings. *Acad. Manage. Rev.* 12 637–647. 10.5465/AMR.1987.4306715

[B23] Conger J. A. Kanungo R. N. Menon S. T. (2000). Charismatic leadership and follower effects. *J. Organ. Behav.* 21 747–767. 10.1002/1099-1379(200011)21:7<747::AID-JOB46>3.0.CO;2-J

[B24] Côté S. Hideg I. (2011). The ability to influence others via emotion displays: a new dimension in emotional intelligence. *Organ. Psychol. Rev.* 1 53–71. 10.1177/2041386610379257

[B25] Dahling J. J. Perez L. A. (2010). Older worker, different actor? Linking age and emotional labor strategies. *Pers. Individ. Dif.* 48 574–578. 10.1016/j.paid.2009.12.009

[B26] Dansereau E. Graen G. Haga W. J. (1975). A vertical dyad linkage approach to leadership within formal organizations: a longitudinal investigation of the role making process. *Organ. Behav. Hum. Perform.* 13 46–78. 10.1016/0030-5073(75)90005-7

[B27] Dasborough M. T. Ashkanasy N. M. (2002). Emotion and attribution of intentionality in leader–member relationships. *Leadersh. Q.* 13 615–634. 10.1016/S1048-9843(02)00147-9

[B28] Dasborough M. T. Ashkanasy N. M. Tee E. Y. J. Tse H. H. M. (2009). What goes around comes around: how meso-level negative emotional contagion can ultimately determine organizational attitudes towards leaders. *Leadersh. Q.* 20 571–585. 10.1016/j.leaqua.2009.04.009

[B29] De Rivera J. (1992). “Emotional climate: social structure and emotional dynamics,” in *International Review of Studies on Emotions*, ed. Strongman K. T. (Chichester: Wiley), 197–218.

[B30] DeChurch L. A. Hiller N. J. Murase T. Doty D. Salas E. (2010). Leadership across levels: levels of leaders and their levels of impact. *Leadersh. Q.* 21 1069–1085. 10.1016/j.leaqua.2010.10.009

[B31] Diefendorff J. M. Greguras G. J. (2009). Contextualizing emotional display rules: examining the roles of targets and discrete emotions in shaping display rule perceptions. *J. Manag.* 35 880–898. 10.1177/0149206308321548

[B32] Diefendorff J. M. Croyle M. H. Gosserand R. H. (2005). The dimensionality and antecedents of emotional labor strategies. *J. Vocat. Behav.* 66 339–357. 10.1016/j.jvb.2004.02.001

[B33] Diefendorff J. M. Erickson R. J. Grandey A. A. Dahling J. (2011). Emotional display rules as work unit norms: a multilevel analysis of emotional labor among nurses. *J. Occup. Health Psychol.* 16 379–392. 10.1037/a0021725 21244168

[B34] Dutton J. E. Dukerich J. M. Harquail C. V. (1994). Organizational images and member identification. *Adm. Sci. Q.* 39 239–263. 10.2307/2393235

[B35] Eberly M. B. Fong C. T. (2013). Leading via the heart and mind: the roles of leader and follower emotions, attributions, and interdependence. *Leadersh. Q.* 24 696–711. 10.1016/j.leaqua.2013.05.003

[B36] Emrich C. G. Brower H. H. Feldman J. M. Garland H. (2001). Images in words: presidential rhetoric, charisma, and greatness. *Adm. Sci. Q.* 46 527–557. 10.2307/3094874

[B37] Fisher C. D. (2000). Mood and emotions while working: missing pieces of job satisfaction? *J. Organ. Behav.* 21 185–202. 10.1002/(SICI)1099-1379(200003)21:2<185::AID-JOB34>3.0.CO;2-M

[B38] Fisher C. D. (2002). Antecedents and consequences of real-time affective reactions at work. *Motiv. Emot.* 26 3–30. 10.1023/A:1015190007468

[B39] Fredrickson B. L. (2001). The role of positive emotions in positive psychology: the broaden-and-build theory of positive emotions. *Am. Psychol.* 56 218–226. 10.1037/0003-066X.56.3.218 11315248 PMC3122271

[B40] Frijda N. H. (1993). “Moods, emotion episodes, and emotions,” in *Handbook of Emotions*, 3 Edn, eds Lewis M. Haviland-Jones J. M. Barrett L. F. (New York, NY: Guilford Press), 381–403.

[B41] Frijda N. H. (2007). *The Laws of Emotion.* Mahwah, NJ: Lawrence Erlbaum Associates, Publishers.

[B42] Gardner W. L. Fischer D. Hunt J. G. (2009). Emotional labor and leadership: a threat to authenticity? *Leadersh. Q.* 20 466–482. 10.1016/j.leaqua.2009.03.011

[B43] Gasper K. Clore G. L. (2000). Do you have to pay attention to your feelings to be influenced by them? *Pers. Soc. Psychol. Bull.* 26 698–711. 10.1177/0146167200268005

[B44] Geertz C. (1973). *The Interpretation of Cultures: Selected Essays*, Vol. 5019 New York, NY: Basic Books, Inc

[B45] George J. M. (1990). Personality, affect, and behaviour in groups. *J. Appl. Psychol.* 75 107–116. 10.1037/0021-9010.75.2.107

[B46] George J. M. (1996). “Group affective tone,” in *Handbook of Work Group Psychology*, ed. West M. (Chichester: Wiley), 77–93.

[B47] George J. M. (2000). Emotions and leadership: the role of emotional intelligence. *Hum. Relat.* 53 1027–1055. 10.1177/0018726700538001

[B48] George J. M. Bettenhausen K. (1990). Understanding prosocial behaviour, sales performance, and turnover: a group-level analysis in a service context. *J. Appl. Psychol.* 75 698–709. 10.1037/0021-9010.75.6.698

[B49] Gerstner C. R. Day D. V. (1997). Meta-analytic review of leader-member exchange theory: correlates and construct ideas. *J. Appl. Psychol.* 82 827–844. 10.1037/0021-9010.82.6.827

[B50] Gioia D. A. Chittipeddi K. (1991). Sensemaking and sensegiving in strategic change initiation. *Strateg. Manage. J.* 12 433–448. 10.1002/smj.4250120604

[B51] Glomb T. M. Tews M. J. (2004). Emotional labor: a conceptualization and scale development. *J. Vocat. Behav.* 64 1–23. 10.1016/S0001-8791(03)00038-1

[B52] Gohm C. L. (2003). Mood regulation and emotional intelligence: individual differences. *J. Pers. Soc. Psychol.* 84 594–607. 10.1037/0022-3514.84.3.594 12635919

[B53] Gohm C. L. Clore G. L. (2002). Four latent traits of emotional experience and their involvement in well-being, coping, and attributional style. *Cogn. Emot.* 16 495–518. 10.1080/02699930143000374

[B54] Gooty J. Connelly S. Griffith J. Gupta A. (2010). Leadership, affect and emotions: a state of the science review. *Leadersh. Q.* 21 979–1004. 10.1016/j.leaqua.2010.10.005

[B55] Grandey A. (2000). Emotion regulation in the workplace: a new way to conceptualize emotional labor. *J. Occup. Health Psychol.* 5 95–110. 10.1037/1076-8998.5.1.95 10658889

[B56] Grandey A. A. Diefendorff J. M. Rupp D. E. (eds) (2013). *Emotional Labor in the 21st Century: Diverse Perspectives on Emotion Regulation at Work.* New York, NY: Routledge.

[B57] Grandey A. A. Rupp D. E. Brice W. N. (2015). Emotional labor threatens decent work: a proposal to eradicate emotional display rules. *J. Organ. Behav.* 36 770–785. 10.1002/job.2020

[B58] Griffith J. Connelly S. Thiel C. Johnson G. (2015). How outstanding leaders lead with affect: an examination of charismatic, ideological, and pragmatic leaders. *Leadersh. Q.* 26 502–517. 10.1016/j.leaqua.2015.03.004

[B59] Gross J. J. (1998). The emerging field of emotion regulation: an integrative review. *Rev. Gen. Psychol.* 2 271–299. 10.1037/1089-2680.2.3.271

[B60] Gross J. J. (2006). *Handbook of Emotion Regulation.* New York, NY: Guilford Press.

[B61] Halverson S. K. Murphy S. E. Riggio R. E. (2004). Charismatic leadership in crisis situations: a laboratory investigation of stress and crisis. *Small Group Res.* 35 495–514. 10.1177/1046496404264178

[B62] Hannah S. T. Luthans F. (2008). “A cognitive affective processing explanation of positive leadership: toward theoretical understanding of the role of psychological capital,” in *Affect and Emotion: New Directions in Management Theory and Research*, ed. Humphrey R. H. (Charlotte, NC: Information Age Publishing).

[B63] Hatfield E. Cacioppo J. T. Rapson R. L. (1994). *Emotional Contagion.* New York, NY: Cambridge University Press.

[B64] Hochschild A. R. (1983). *The Managed Heart: Commercialization of Human Feeling.* Berkeley, CA: University of California Press.

[B65] Hoerger M. Chapman B. P. Epstein R. M. Duberstein P. R. (2012). Emotional intelligence: a theoretical framework for individual differences in affective forecasting. *Emotion* 12 716–725. 10.1037/a0026724 22251053 PMC3330168

[B66] Hoffman B. J. Woehr D. J. Maldagen-Youngjohn R. Lyons B. D. (2011). Great man or great myth? A quantitative review of the relationship between individual differences and leader effectiveness. *J. Occup. Organ. Psychol.* 84 347–381.

[B67] Hulsheger U. R. Schewe A. F. (2011). On the costs and benefits of emotional labor. A meta-analysis of three decades of research. *J. Occup. Health Psychol.* 16 361–389. 10.1037/a0022876 21728441

[B68] Humphrey R. H. (2002). The many faces of emotional leadership. *Leadersh. Q.* 13 493–504. 10.1016/S1048-9843(02)00140-6

[B69] Humphrey R. H. (2008). “The right way to lead with emotional labor,” in *Affect and Emotion: New Directions in Management Theory and Research*, ed. Humphrey R. H. (Charlotte, NC: Information Age), 1–17.

[B70] Humphrey R. H. (2012). How do leaders use emotional labor? *J. Organ. Behav.* 33 740–744. 10.1002/job.1791

[B71] Humphrey R. H. (2013). *Effective Leadership: Theories, Cases, and Applications.* Los Angeles, CA: SAGE Publications.

[B72] Humphrey R. H. Ashforth B. E. Diefendorff J. M. (2015). The bright side of emotional labor. *J. Organ. Behav.* 36 749–769. 10.1002/job.2019

[B73] Humphrey R. H. Pollack J. M. Hawver T. (2008). Leading with emotional labor. *J. Manag. Psychol.* 23 151–168. 10.1108/02683940810850790

[B74] Hunt J. G. Gardner W. L. Fischer D. (2008). “Leader emotional displays from near and far: the implications of close versus distant leadership for leader emotional labor and authenticity,” in *Affect and Emotion: New Directions in Management Theory and Research*, ed. Humphrey R. H. (Charlotte, NC: Information Age), 43–65.

[B75] Ilies R. Morgeson F. P. Nahrang J. D. (2005). Authentic leadership and eudaemonic well-being: understanding leader-follower outcomes. *Leadersh. Q.* 16 373–394. 10.1016/j.leaqua.2005.03.002

[B76] Ilies R. Nahrgang J. D. Morgeson F. P. (2007). Leader–member exchange and citizenship behaviors: a meta-analysis. *J. Appl. Psychol.* 92 269–277. 10.1037/0021-9010.92.1.269 17227168

[B77] Iszatt-White M. (2009). Leadership as emotional labour: the effortful accomplishment of valuing practices. *Leadership* 5 447–467. 10.1177/1742715009343032

[B78] Iszatt-White M. (2012). “Leadership as emotional labour: so what’s new?,” in *Leadership as Emotional Labour: Management and the “Managed Heart”*, ed. Iszatt-White M. (Abingdon: Routledge), 14–36.

[B79] Izard C. E. (1991). *The Psychology of Emotions.* Berlin: Springer Science & Business Media.

[B80] Izard C. E. (2009). Emotion theory and research: highlights, unanswered questions, and emerging issues. *Annu. Rev. Psychol.* 60 1–25. 10.1146/annurev.psych.60.110707.163539 18729725 PMC2723854

[B81] Johnson S. K. (2008). I second that emotion: effects of emotional contagion and affect at work on leader and follower outcomes. *Leadersh. Q.* 19 1–19. 10.1016/j.leaqua.2007.12.001

[B82] Judge T. A. Bono J. E. Ilies R. Gerhardt M. W. (2002). Personality and leadership: a qualitative and quantitative review. *J. Appl. Psychol.* 87 765–780. 10.1037/0021-9010.87.4.765 12184579

[B83] Kacmar K. M. Bachrach D. G. Harris K. J. Noble D. (2012). Exploring the role of supervisor trust in the associations between multiple sources of relationship conflict and organizational citizenship behaviour. *Leadersh. Q.* 23 43–54. 10.1016/j.leaqua.2011.11.004

[B84] Kellett J. B. Humphrey R. H. Sleeth R. G. (2009). Career development, collective efficacy, and individual task performance. *Career Dev. Int.* 14 534–546. 10.1108/13620430910997286

[B85] Kemper T. D. (1990). “Social relations and emotions: a structural approach,” in *Research Agendas in the Sociology of Emotions*, ed. Kemper T. D. (Albany, NY: State University of New York System), 207–237.

[B86] Kemper T. D. (1991). Predicting emotions from social relations. *Soc. Psychol. Q.* 54 330–342. 10.2307/2786845

[B87] Koning L. F. Van Kleef G. A. (2015). How leaders’ emotional displays shape followers’ organizational citizenship behavior. *Leadersh. Q.* 26 489–501. 10.1016/j.leaqua.2015.03.001

[B88] Liden R. C. Maslyn J. M. (1998). Multidimensionality of leader-member exchange: an empirical assessment through scale development. *J. Manag.* 24 43–72. 10.1177/014920639802400105

[B89] Lu X. Guy M. E. (2014). How emotional labor and ethical leadership affect job engagement for Chinese public servants. *Public Pers. Manage.* 43 3–24. 10.1177/0091026013512278

[B90] Luthans F. Avolio B. J. Avey J. B. Norman S. M. (2007). Positive psychological capital: measurement and relationship with performance and satisfaction. *Pers. Psychol.* 60 541–572. 10.1111/j.1744-6570.2007.00083.x

[B91] Lyubomirsky S. (2008). *The How of Happiness: A Scientific Approach to Getting the Life You Want.* New York, NY: Penguin Press.

[B92] Madera J. M. Smith D. B. (2009). The effect of leader negative emotions on evaluations of leadership in a crisis situation: the role of anger and sadness. *Leadersh. Q.* 20 103–114. 10.1016/j.leaqua.2009.01.007

[B93] Maitlis S. (2005). The social processes of organizational sensemaking. *Acad. Manage. J.* 48 21–49. 10.5465/AMJ.2005.15993111

[B94] Marroquín B. Boyle C. C. Nolen-Hoeksema S. Stanton A. L. (2016). Using emotion as information in future-oriented cognition: individual differences in the context of state negative affect. *Pers. Individ. Dif.* 95 121–126. 10.1016/j.paid.2016.02.033 27041783 PMC4811627

[B95] Mauss I. B. Robinson M. D. (2009). Measures of emotions: a review. *Cogn. Emot.* 23 209–237. 10.1080/02699930802204677 19809584 PMC2756702

[B96] Menges J. I. Kilduff M. (2015). Group emotions: cutting the Gordian knots concerning terms, levels of analysis, and processes. *Acad. Manage. Ann.* 9 845–928. 10.1080/19416520.2015.1033148

[B97] Mio J. S. Riggio R. E. Levin S. Reese R. (2005). Presidential leadership and charisma: the effects of metaphor. *Leadersh. Q.* 16 287–294. 10.1016/j.leaqua.2005.01.005

[B98] Murphy S. E. Ensher E. A. (2008). A qualitative analysis of charismatic leadership in creative teams: the case of television directors. *Leadersh. Q.* 19 335–352. 10.1016/j.leaqua.2008.03.006

[B99] Naidoo L. J. Lord R. G. (2008). Speech imagery and perceptions of charisma: the mediating role of positive affect. *Leadersh. Q.* 19 283–296.

[B100] Newcombe M. J. Ashkanasy N. M. (2002). The role of affect and affective congruence in perceptions of leaders: an experimental study. *Leadersh. Q.* 13 601–614. 10.1016/S1048-9843(02)00146-7

[B101] Niedenthal P. M. Brauer M. (2012). Social functionality of human emotion. *Annu. Rev. Psychol.* 63 259–285. 10.1146/annurev.psych.121208.131605 22017377

[B102] Ortony A. Clore G. L. Collins A. (1988). *The Cognitive Structure of Emotions*, New York, NY: Cambridge University Press.

[B103] Pescosolido A. T. (2002). Emergent leaders as managers of group emotion. *Leadersh. Q.* 13 583–599. 10.1016/S1048-9843(02)00145-5

[B104] Pirola-Merlo A. Härtel C. E. J. Mann L. Hirst G. (2002). How leaders influence the impact of affective events on team climate and performance in R&D teams. *Leadersh. Q.* 13 561–581. 10.1016/S1048-9843(02)00144-3

[B105] Pratt M. G. Rafaeli A. (1997). Organizational dress as a symbol of multilayered social identities. *Acad. Manage. J.* 40 862–898. 10.2307/256951

[B106] Pratt M. G. Rafaeli A. (2001). Symbols as a language of organizational relationships. *Res. Organ. Behav.* 23 93–132. 10.1016/S0191-3085(01)23004-4

[B107] Rafaeli A. Sutton R. I. (1987). Expression of emotion as part of the work role. *Acad. Manage. Rev.* 12 23–37. 10.2307/257991

[B108] Rafaeli A. Sutton R. I. (1989). The expression of emotion in organizational life. *Res. Organ. Behav.* 11 1–42.

[B109] Rafaeli A. Worline M. (2001). Individual emotion in work organizations. *Soc. Sci. Inform.* 40 95–123. 10.1177/053901801040001006

[B110] Rajah R. Song Z. Arvey R. D. (2011). Emotionality and leadership: taking stock of the past decade of research. *Leadersh. Q.* 22 1107–1119. 10.1016/j.leaqua.2011.09.006

[B111] Schaubroeck J. M. Shao P. (2012). The role of attribution in how followers respond to the emotional expression of male and female leaders. *Leadersh. Q.* 23 27–42. 10.1016/j.leaqua.2011.11.003

[B112] Schneider A. Gardner W. L. Hinojosa A. Marin A. (2014). Emotional responses of leaders to passive versus active members. *Leadership* 10 412–436. 10.1177/1742715013504424

[B113] Smircich L. Morgan G. (1982). Leadership: the management of meaning. *J. Appl. Behav. Sci.* 18 257–273. 10.1177/002188638201800303 10260212

[B114] Schriesheim C. A. Castro S. Cogliser C. C. (1999). Leader-member exchange (LMX) research: a comprehensive review of theory, measurement, and data-analytic practices. *Leadersh. Q.* 10 63–113. 10.1016/S1048-9843(99)80009-5

[B115] Sutton R. L. Rafaeli A. (1988). Untangling the relationship between displayed emotions and organizational sales: the case of convenience stores. *Acad. Manage. J.* 32 481–487.

[B116] Sy T. Côté S. Saavedra R. (2005). The contagious leader: impact of the leader’s mood on the mood of group members, group affective tone, and group processes. *J. Appl. Psychol.* 90 295–305. 10.1037/0021-9010.90.2.295 15769239

[B117] Tee E. Y. J. (2015). The emotional link: leadership and the role of implicit and explicit emotional contagion processes across multiple organizational levels. *Leadersh. Q.* 26 654–670. 10.1016/j.leaqua.2015.05.009

[B118] Tiedens L. Z. Ellsworth P. C. Mesquita B. (2000). Stereotypes about sentiments and status: emotional expectations for high- and low-status group members. *Pers. Soc. Psychol. Bull.* 26 560–575. 10.1177/0146167200267004

[B119] Van Kleef G. A. (2009). How emotions regulate social life: the emotions as social information (EASI) model. *Curr. Dir. Psychol. Sci.* 18 184–188. 10.1111/j.1467-8721.2009.01633.x

[B120] Van Kleef G. A. Homan A. C. Beersma B. Van Knippenberg D. Van Knippenberg B. Damen F. (2009). Searing sentiment or cold calculation? The effects of leader emotional displays on team performance depend on follower epistemic motivation. *Acad. Manage. J.* 52 562–580. 10.5465/AMJ.2009.41331253

[B121] Van Kleef G. A. Van Doorn E. A. Heerdink M. W. Koning L. F. (2011). Emotion if for influence. *Eur. Rev. Soc. Psychol.* 22 114–163. 10.1080/10463283.2011.627192

[B122] Vijayalakshmi V. Bhattacharyya S. (2012). Emotional contagion and its relevance to individual behavior and organizational processes: a positional paper. *J. Bus. Psychol.* 22 363–374. 10.1007/s10869-011-9243-4

[B123] Visser V. A. van Knippenberg D. van Kleef G. A. Wisse B. (2013). How leader displays of happiness and sadness influence follower performance: emotional contagion and creative versus analytical performance. *Leadersh. Q.* 24 172–188. 10.1016/j.leaqua.2012.09.003

[B124] Walter F. Cole M. S. Humphrey R. H. (2011). Emotional intelligence: sine qua non of leadership or folderol? *Acad. Manage. Perspect.* 25 45–59. 10.5465/AMP.2011.59198449

[B125] Wang G. Seibert S. E. Boles T. L. (2011). “Synthesizing what we know and looking ahead: a meta-analytical review of 30 years of emotional labor research,” in *What Have We Learned? Ten Years (Research on Emotion in Organizations)*, Chap. 1 Vol. 7 eds Härtel C. E. J. Ashkanasy N. M. Zerbe W. J. (Bingley: Emerald Group Publishing Limited), 15–43.

[B126] Watson D. Tellegen A. (1985). Toward a consensual structure of mood. *Psychol. Bull.* 98 219–235. 10.1037/0033-2909.98.2.219 3901060

[B127] Weiss H. M. Cropanzano R. (1996). “Affective events theory: a theoretical discussion of the structure, causes, and consequences of affective experiences at work,” in *Research in Organizational Behavior* Vol. 18 eds Staw B. M. Cummings L. L. (Greenwich, CT: JAI Press), 1–74.

[B128] Weiss H. M. Nichols J. P. Daus C. S. (1999). An examination of the joint effects of affective experiences and job beliefs on job satisfaction and variations in affective experiences over time. *Organ. Behav. Hum. Decis. Process.* 78 1–24. 10.1006/obhd.1999.2824 10092469

[B129] Zampetakis L. A. Moustakis V. (2011). Managers’ trait emotional intelligence and group outcomes: the case of group job satisfaction. *Small Group Res.* 42 77–102. 10.1177/1046496410373627

